# Effect of an herbal/botanical supplement on strength, balance, and muscle function following 12-weeks of resistance training: a placebo controlled study

**DOI:** 10.1186/1550-2783-11-23

**Published:** 2014-05-28

**Authors:** Jonathan Furlong, Corey A Rynders, Mark Sutherlin, James Patrie, Frank I Katch, Jay Hertel, Arthur Weltman

**Affiliations:** 1 Department of Kinesiology Exercise Physiology Core Laboratory, University of Virginia, 203 Memorial Gymnasium, Charlottesville 22904, Virginia, USA; 2 Department of Human Movement Sciences, Human Performance Laboratory, Old Dominion University, Norfolk, VA, USA

**Keywords:** Anti-oxidant, Blue-green Algae, Strength exercise

## Abstract

**Background:**

StemSport (SS; StemTech International, Inc. San Clemente, CA) contains a proprietary blend of the botanical Aphanizomenon flos-aquae and several herbal antioxidant and anti-inflammatory substances. SS has been purported to accelerate tissue repair and restore muscle function following resistance exercise. Here, we examine the effects of SS supplementation on strength adaptations resulting from a 12-week resistance training program in healthy young adults.

**Methods:**

Twenty-four young adults (16 males, 8 females, mean age = 20.5 ± 1.9 years, mass = 70.9 ± 11.9 kg, stature = 176.6 ± 9.9 cm) completed the twelve week training program. The study design was a double-blind, placebo controlled parallel group trial. Subjects either received placebo or StemSport supplement (SS; mg/day) during the training. 1-RM bench press, 1-RM leg press, vertical jump height, balance (star excursion and center of mass excursion), isokinetic strength (elbow and knee flexion/extension) and perception of recovery were measured at baseline and following the 12-week training intervention.

**Results:**

Resistance training increased 1-RM strength (p < 0.008), vertical jump height (p < 0.03), and isokinetic strength (p < 0.05) in both SS and placebo groups. No significant group-by-time interactions were observed (all p-values >0.10).

**Conclusions:**

These data suggest that compared to placebo, the SS herbal/botanical supplement did not enhance training induced adaptations to strength, balance, and muscle function above strength training alone.

## Introduction

Various nutritional supplements have been investigated for accelerating recovery from resistance exercise. For example, carbohydrate ingestion within 1 to 2 hours following a strength training session promotes glycogen re-synthesis and decreases muscle recovery time [1,2]. Protein supplementation stimulates protein synthesis, which may aid recovery, thus leading to enhanced strength gains with resistance training [3,4]. Several herbal supplements with anti-inflammatory and/or anti-oxidant properties also purport to enhance recovery from resistance exercise and enhance strength gains. There is no consensus in the literature concerning how herbal supplements impact the magnitude of their performance enhancing benefits [5].

We recently examined the effects of a dietary supplement containing a blend of herbal antioxidants/anti-inflammatory substances including the fresh water blue-green algae Aphanizomenon flos-aquae (StemSport; SS, StemTech International, Inc. San Clemente, CA) on the severity and time course of delayed onset muscle soreness (DOMS) following an acute bout of eccentric upper arm exercise (Rynders et al., In Review, JISSN). Our study reported that compared to a placebo, SS supplementation had no effect on muscle swelling, isometric strength, muscle pain and tenderness, and swelling measured 24 h, 48 h, 72 h, and 168 h (1 week) post-eccentric exercise (Rynders et al., In Review, JISSN). There were no differences in measures of recovery between SS and placebo after DOMS, yet it is possible that the amount of muscle tissue damage elicited by the DOMS protocol negated any beneficial effect of the supplement. If a less dramatic overload were utilized such as strength training, it is possible that the supplement would enhance recovery and performance in a subsequent exercise bout. This would lead to a greater cumulative training response (i.e. greater total work completed per workout session).

The present placebo-controlled study examined the effects of SS supplementation on the adaptations to strength, balance, and muscle function resulting from a 12-week resistance training program in healthy young adults. We hypothesized that SS would accelerate the rate of recovery from each training session, allowing for a greater overload in subsequent training sessions, and an enhanced training response.

## Methods

### Experimental approach to the problem

This was a randomized, double blind, placebo-controlled, parallel group design to examine the effects of SS supplementation on training adaptations following a 12-week resistance training program. Independent variables included supplement type (SS or Placebo) and measurement period (pre- and post- 12 weeks of training). Dependent variables included 1 repetition max (1RM) bench press and 1RM leg press; vertical jump; Star Excursion Balance Test (SEBT); static balance; isokinetic concentric phase peak torque and average power for knee and elbow flexion and extension; and perception of recovery.

### Subjects

The University Institutional Review Board approved the study and subjects provided written informed consent prior to participation. Thirty-five healthy male and female undergraduate and graduate students were recruited from Lifetime Physical Activity weight training classes. All participants were enrolled in an introductory strength training class, and had not participated in more than 1 day/week of resistance training prior to study enrollment. All participants provided written informed consent and a medical history. Exclusion criteria included a history of kidney disease, vascular disease, circulatory insufficiencies, or cancer; use of anti-depressants, warfarin, or any protein/muscle building supplements; and self-reported pregnancy, drug use, or smoking.

### SS and placebo supplementation

Subjects were randomly assigned to receive either the active SS supplement (n = 17) or placebo (n = 18). The SS ingredient list is presented in Table 1. Subjects were instructed to adhere to the following dosing schedule according to manufacturer recommendations: 1000 mg of Aphanizomenon flos-aquae extract 3 times per day (breakfast, lunch, and dinner) and 1575 mg of a proprietary herbal/botanical blend twice per day (breakfast and dinner). One additional dose of Aphanizomenon flos-aquae and one additional dose of the herbal/botanical blend were consumed before exercise and after exercise according to manufacture instructions. The placebo consisted of 1000 mg of encapsulated corn starch. Subjects were required to maintain a pill diary throughout the study and were instructed to forfeit any capsules not ingested during the study period. Supplements (SS and placebo) were dispensed weekly by the University investigational pharmacy. Over-the-counter analgesic and anti-inflammatory medications (i.e. Tylenol, Advil, Ibuprofen, Motrin, Bextra, Celebrex, etc.) were prohibited during the supplementation period.

**Table 1:** StemSport ingredient list and purported benefits

**Ingredient**	**Amount per serving**	**Purported benefit**		
**1. Aphanizomenon flos-aquae extract**	1000mg	Increase the number of circulating stem cells		
**2. Proprietary herbal/botanical blend**	1575mg			
Cats claw	--	Antioxidant		
Mangosteen	--	Antioxidant		
Rehmannia	--	Anti-inflammatory		
Berry extracts	--	Antioxidant		
Nattokinase	--	Anti-inflammatory/fibrinolytic		
Serrapeptase	--	Anti-inflammatory/fibrinolytic		
Curcumin	--	Antioxidant/anti-inflammatory		

Two subjects in the SS condition and one subject in the placebo condition withdrew prior to beginning the training intervention. Five subjects in the SS condition withdrew during the 12-week training program due to injury (n = 1), an adverse reaction to the supplement (n = 1), or time constraints (n = 3). Three subjects in the placebo condition withdrew during the intervention period due to time constraints. Twenty-four subjects completed the twelve week training program.

### Procedures

Pre- and post-training (12-weeks) testing consisted of 1RM bench press and 1RM leg press, isokinetic concentric phase peak torque and average power for knee and elbow flexion and extension, vertical jump, SEBT, and static balance.

#### 1RM testing

The IRM testing was performed using the National Strength and Conditioning Association ARM protocol [6]. Participants began the 1RM bench press and leg press assessments by warming up with repetitions on the bench press using a 20.5-kg bar and free weights or dumbbells, and multiple repetitions on the leg press machine (Hammer Strength, Schiller Park, IL, USA). The goal was to build to the 1RM load within five attempts. For the bench press, a successful repetition was scored if the weight was lowered to the chest and raised to full arm extension without losing foot, hip, back, or shoulder contact with the bench of the floor without help provided by a spotter. For the leg press, a successful repetition was scored if the weight was lowered such that knees created a 90° angle and raised to full leg extension without the subject losing back or shoulder contact with the machine and without spotter assistance. Two failed attempts at a given weight or voluntary termination ended each test.

#### Isokinetic strength testing

A Biodex System 3 multijoint dynamometer (Shirley, NY) was used for isokinetic assessments. Subjects performed 3 sets of 5 repetitions for each of the isokinetic exercises, with a 90 second rest interval between sets. Verbal encouragement was given for each repetition, and testing was preceded with 10 to 15 practice repetitions to familiarize the subject with the isokinetic device. Each exercise was conducted at angular velocities of 60 and 180 degrees per second (deg.sec^−1^). The isokinetic knee flexion and extension tests were performed from full knee extension (0°) to 90° flexion. The isokinetic elbow flexion and extension tests were performed from full elbow extension (0°) to 160° of flexion. For all tests, the seatback angle was set at 85°, and the hips were in 90° of flexion. For each motion, peak torque and average power from the 3 sets were averaged prior to statistical analysis.

#### Static balance

Static balance was assessed using an Accusway force plate (AMTI, Watertown, MA). Subjects performed three trials of single-limb stance on their dominant leg with eyes open and then closed for 15 seconds. Subjects were instructed to stand as still as possible with arms folded across their chest, holding the opposite limb at 45° of knee flexion and 30° of hip flexion. If the subject touched down with the non-stance limb, made contact with the stance limb, or was unable to maintain standing posture during the 15-s trial, the trial was terminated and repeated. Traditional center of pressure (COP) based measures of the mean velocity of COP excursions (total COP excursion length in centimeters divided by the 10-s trial time) and the area of the 95% confidence ellipse of COP excursions were calculated via computer software. The mean of each measure for the three eyes-open and eyes-closed trials were used for statistical analysis.

#### Star excursion balance test

A trained investigator assessed anterior, posteromedial, and posterolateral components of the SEBT. Subjects maintained single limb stance on the test limb while reaching as far as possible with the contralateral limb in the given direction, made a light touch on the line at their point of maximum reach, and returned to the starting position. Subjects performed 5 practice trials in each reach direction. The reach distances of three trials in each direction were recorded. Trials were repeated if a subject bore excessive weight on the reaching limb, removed the stance foot from the starting position, or lost balance. Reach distance were normalized to subject leg length in accordance to previously established methods using the mean of three trials for each direction [7].

#### Vertical jump

Subjects performed three trials of a counter-movement vertical jump using a Vertec Jump Measurement System (JumpUSA, Sunnyvale, CA). The highest attained value was used for analysis.

#### Training intervention

Subjects performed supervised resistance training exercises 3 times a week for 12 weeks. Subjects performed 2 sets of 10 exercises using a combination of free weights and machines. When the subject was able to successfully perform 2 sets of 10 repetitions for an exercise, the weight was increased by 5 to 25 pounds at the next training session. The same 10 exercises were performed each training session for 4 weeks, and then modified (i.e. lunges to split squats). Examples of exercises performed included bench press, leg press, seated row, overhead press, knee extension, hamstring curls, biceps curls, triceps extensions, and lunges, calf raises. Subjects maintained training logs, recording the weights and repetitions completed during each session.

#### Perception of recovery

Perception of recovery from strength training was assessed using a visual analog scale throughout the 12-week training program at weeks 1, 2, 4, 6, 8, 10, and 12. Subjects were instructed to make a vertical line at the position on the scale to represent their perceived recovery from training, with the left end point labeled “completely recovered” and the right end point “not recovered at all”. The measured distance of the marked position from the left end point served as the score and normalized by dividing by total scale length.

### Statistical analyses

Data were evaluated for normality using the Shapiro-Wilk Test. Variables that violated the normality assumption (Shapiro-Wilk p-value < 0.05) were log transformed for analysis. Separate 2-factor analysis of variance (ANOVA) with repeated measures over time was executed with the treatment group (SS or placebo) as the independent variable. For the performance tests, the dependent variable was the respective outcome measure. Thus, there was 1 between-subjects factor (treatment group) and 1 within-subjects factor (time). For the perception of recovery scale, the dependent variable was the normalized score calculated as the distance from the left endpoint divided by the total length of the scale. Scales were completed at weeks 1, 2, 4, 6, 8, 10, and 12; thus there was 1 between-subjects factor (treatment group) and 7 within-subjects factors. Where significant main effects were observed, post hoc procedures were applied to examine within group changes over time. Independent samples t-tests were conducted to examine differences in adherence to training, where the number of training sessions completed served as the dependent variable, and the percentage of pills consumed to verify adherence to supplement consumption. The threshold for significance for all tests was set at p < 0.05.

## Results

### Adherence to training

There was no significant difference between groups in adherence to training assessed by the number of training sessions completed (30.3 sessions for placebo, 29.8 sessions for SS, p = 0.50), or adherence to treatment assessed by the percentage of pills ingested (92.9% of pills in placebo, 86.3% of pills in SS, p = 0.10).

### 1-RM

**Figure 1:**
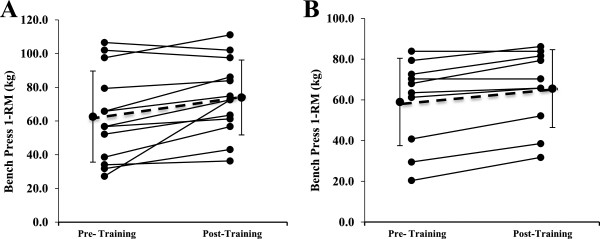
**Individual and mean (±SD) responses in 1RM bench press in (A) placebo condition and (B) StemSport condition.** Both groups improved significantly with training (*p <* 0.01), but there was no time × condition interaction (*p =* 0.28).

**Figure 2:**
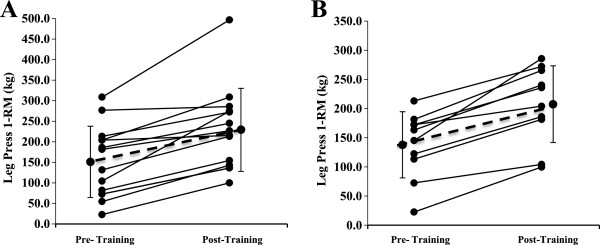
**Individual and mean (±SD) responses in 1RM leg press in (A) placebo condition and (B) StemSport condition.** Both groups improved significantly with training (*p* < 0.001), but there was no time × condition interaction (*p* = 0.652).

Figures 1 and 2 display the individual and mean responses for 1 RM bench press and 1 RM leg press. Bench press 1-RM increased by 18.2% (p = 0.008) with placebo and 11.0% with S (p = 0.001). Leg press 1-RM increased by 48.6% with placebo (p < 0.001) and by 50.5% with SS (p < 0.001). There were no differences in 1-RM improvement (bench press and leg press) between placebo and SS conditions (p-values > 0.28). Similar results were observed when the values were normalized for body weight (data shown in Table 2).

**Table 2:** Mean (±SD) pre- and post-training values for strength, balance, and muscle function in the StemSport and Placebo supplementation conditions

**Parameter**	**StemSport**		**Placebo**	
	**Pre**	**Post**	**Pre**	**Post**
Weight Adjusted Bench Press 1RM*	0.84 ± 0.25	0.95 ± 0.21	0.83 ± 0.28	1.00 ± 0.22
Weight Adjusted Leg Press 1RM*	1.95 ± 0.71	2.97 ± 0.64	2.10 ± 0.85	3.19 ± 0.94
Height Adjusted Vertical Jump*	0.28 ± 0.06	0.31 ± 0.06	0.27 ± 0.04	0.29 ± 0.04
Anterior SEBT	0.70 ± 0.11	0.70 ± 0.07	0.71 ± 0.07	0.68 ± 0.06
Posteromedial SEBT	0.91 ± 0.10	0.91 ± 0.60	0.92 ± 0.10	0.89 ± 0.09
Posterolateral SEBT	0.86 ± 0.11	0.86 ± 0.08	0.87 ± 0.11	0.85 ± 0.10
Eyes Open COM Excursion Velocity (cm/sec)^†^	4.49 ± 1.36	4.50 ± 1.16	4.71 ± 2.02	4.05 ± 1.15
Eyes Open COM Excursion Area	6.24 ± 2.76	5.79 ± 2.82	6.24 ± 2.49	5.40 ± 2.09
Eyes Closed COM Excursion Velocity (cm/sec)	9.91 ± 2.90	10.30 ± 3.33	11.33 ± 3.94	9.65 ± 2.98
Eyes Closed COM Excursion Area	32.85 ± 13.6	33.87 ± 12.0	32.54 ± 11.1	28.28 ± 8.36
Elbow Extension Peak Torque @ 60°/sec (N · m)*	46.79 ± 14.2	51.64 ± 13.4	47.09 ± 14.4	60.04 ± 22.6
Elbow Extension Peak Torque @ 180°/sec (N · m)^†^	30.65 ± 11.7	32.48 ± 9.7	30.65 ± 8.5	34.55 ± 10.5
Elbow Extension Average Power @ 60°/sec (W)^†^	42.82 ± 15.0	46.58 ± 13.1	42.43 ± 13.2	54.68 ± 20.3
Elbow Extension Average Power @ 180°/sec (W)^†^	60.11 ± 28.3	63.58 ± 25.1	54.80 ± 22.0	68.03 ± 25.0
Elbow Flexion Peak Torque @ 60°/sec (N · m)^†^	47.94 ± 11.7	54.98 ± 14.4	48.26 ± 15.6	58.05 ± 20.1
Elbow Flexion Peak Torque @ 180°/sec (N · m)^†^	32.99 ± 8.8	38.35 ± 11.6	32.90 ± 11.9	39.05 ± 13.08
Elbow Flexion Average Power @ 60°/sec (W)*	44.1 ± 11.0	51.05 ± 14.4	45.21 ± 16.1	56.40 ± 20.3
Elbow Flexion Average Power @ 180°/sec (W)	58.27 ± 19.7	68.42 ± 27.0	58.97 ± 31.0	70.09 ± 28.2
Knee Extension Peak Torque @ 60°/sec (N · m)Ω	122.5 ± 32.8	103.9 ± 25.6	124.99 ± 42.8	114.7 ± 44.6
Knee Extension Peak Torque @ 180°/sec (N · m)	83.7 ± 21.5	76.2 ± 15.9	85.24 ± 28.7	74.82 ± 29.5
Knee Extension Average Power @ 60°/sec (W)Ω	101.5 ± 27.6	88.9 ± 21.5	106.4 ± 37.3	94.8 ± 25.5
Knee Extension Average Power @ 180°/sec (W)	157.6 ± 46.9	146.0 ± 30.3	173.3 ± 76.7	139.7 ± 59.9
Knee Flexion Peak Torque @ 60°/sec (N · m)	64.4 ± 14.6	57.1 ± 12.9	71.0 ± 24.8	64.8 ± 24.9
Knee Flexion Peak Torque @ 180°/sec (N · m)	48.2 ± 14.2	45.4 ± 9.4	56.1 ± 21.6	46.9 ± 21.4
Knee Flexion Average Power @ 60°/sec (W)	56.4 ± 15.8	53.5 ± 14.6	66.5 ± 26.6	61.1 ± 24.8
Knee Flexion Average Power @ 180°/sec (W)	89.5 ± 36.7	84.2 ± 23.6	114.0 ± 54.1	92.5 ± 46.2

1-RM = 1 repetition maximum; SEBT = Star excursion balance test; COM = center of mass; kg = kilogram; cm = centimeter; sec = second; N^.^m = newton meter; W = watts. * = Significant improvement with training in both conditions, p < 0.05. † = Significant improvement with training in placebo condition only, p < 0.05. Ω = Significant decrement with training in StemSport condition only, p < 0.05.

### Vertical jump

Vertical jump increased 7.2% with placebo (p = 0.03) and 10.6% with SS (p =0.001), but no significant between group differences (p > 0.05; Table 2).

### Isokinetic strength

Seven of the eight measures of isokinetic elbow flexion and extension strength improved in the placebo condition compared to only two measures in the SS condition (Table 2). No pre- to post-training improvements were observed for the measures of isokinetic knee extension and flexion strength. Post hoc tests revealed decrements in of two of the eight measures of isokinetic knee extension and flexion strength in the SS condition (Table 2).

### Balance

There was a significant improvement in eyes open center of pressure excursion velocity in the placebo condition, but not for SS. SEBT measures did not improve post-training in either group (Table 2).

### Perception of recovery

**Figure 3:**
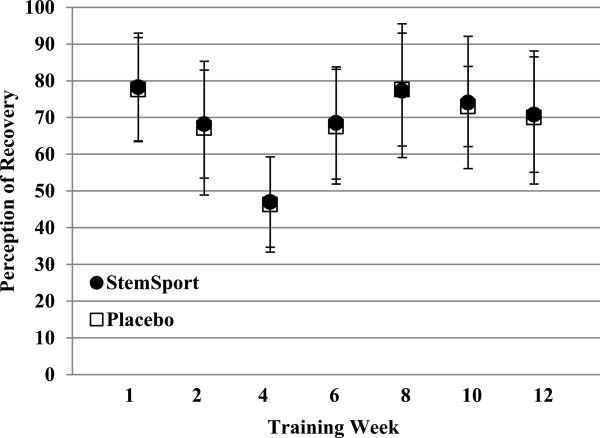
**Weekly mean (±SD) perception of recovery.** ANOVA analyses revealed a significant main effect of time on perception of recovery (*p* < 0.001), but no significant condition × time interaction (*p =* 0.895).

Figure 3 presents the weekly mean perception of recovery scores for both conditions. There was a significant effect of time (p < 0.001) on perception of recovery, but no significant group by time interaction effects (p = 0.895).

## Discussion

The manufacturer of StemSport claims that usage of the product “may play a role in assisting the recovery process, thus reducing recovery time and enhancing the natural renewal process” [8]. In the present study we tested the manufacturer claims and hypothesized that if the claims were accurate, enhanced recovery in response to the SS supplement would improve performance in subsequent exercise training sessions and ultimately lead to a greater cumulative training response and larger strength gains. The major findings of the present study were, 1) twelve weeks of strength training significantly improved muscle strength and function and 2) compared to placebo, SS supplementation did not provide additional benefits above resistance training alone.

To our knowledge, this is the first study evaluating the effects of SS supplementation in response to strength training. SS is a commercially available nutritional product purported to increase the concentration of circulating stem cells, while reducing oxidative and inflammatory stress, which the manufacturers suggest will accelerate post-exercise recovery.

The primary ingredient of SS includes an extract of the fresh water botanical Aphanizomenon flos-aquae (AFA). AFA has been shown to increase the circulating level of human bone marrow derived stem cells [9,10]. Significant increases in the proliferation of cultures of both human bone marrow cells and human CD34+ stem cell with in vitro administration of AFA [10]. In a randomized double-blind placebo controlled crossover study, oral administration of SS produced transient but significant increase in in vivo concentrations of circulating human CD34+ stem cells, peaking at 25% above baseline at 1 hour, with only minor fluctuations observed in a placebo condition [9].

No measurements of circulating stem cells were collected in the present study, and thus the role of stem cells in the recovery from resistance training remains unclear. The supplementation protocol failed to produce any improvements with resistance training above placebo, suggesting that the transient increase in circulating stem cells associated with SS was inadequate to promote accelerated post-exercise recovery. It seems reasonable to suggest that elevated levels of stem cells above those typically observed do not play a significant role in recovery from resistance training, or that SS did not adequately increase circulating stem cells.

StemSport contains a proprietary blend of natural and herbal substances, with documented anti-oxidative, anti-inflammatory, and fibrinolytic effects [11-16]. The manufacturers suggest that by reducing oxidative and inflammatory stress while promoting fibrinolysis, SS would promote post-exercise recovery. No previous studies have examined the effects of SS on recovery from resistance training, although the effects of other anti-oxidative and anti-inflammatory substances on resistance training have been explored [17-19]. Bloomer et al. [17] examined the effects of anti-oxidant supplementation on the acute recovery from an eccentric strength training bout. Anti-oxidant supplementation was not associated with any improvements in blood markers of recovery, perceived muscle soreness, or muscle function. Similarly, no difference in strength gains with vitamin C and E supplementation compared to placebo occurred after 6 months of resistance training in older adults [18]. Antioxidant supplementation may blunt the endogenous adaptive responses to exercise-induced oxidative stress such as improvements in insulin sensitivity [20]. The consequences of these effects remain unclear, yet the limited data demonstrate no ergogenic benefit associated with antioxidant supplementation during resistance training [17,18].

Studies regarding the effects of anti-inflammatory agents on resistance training have focused primarily on non-steroidal anti-inflammatories (NSAIDs). A counter-balanced, double-blind, randomized trial, comparing adaptations to resistance training with ibuprofen supplementation versus placebo in young adults showed no changes in strength or hypertrophy, or in reported muscle soreness [20]. Animal models suggest that the inhibition of cyclo-oxygenase activity associated with NSAIDs may impair muscle hypertrophy [21]. Although not measured in the present study, a prior study using the DOMS model indicated that SS had no effect on circulating inflammatory markers (IL-6 and hsCRP) (Rynders et al. JSCR, In Review).

A secondary finding of the present study demonstrated significant reductions in the perception of recovery from resistance training after 4 weeks, with only minor fluctuations observed throughout the rest of the 12 week period. Flann et al. [22] reported a similar observation in untrained subjects during an eccentric strength training protocol, although their program intentionally utilized a three week “ramp up” period.

An unexpected finding of the present study was the lack of significant change in most measures of knee isokinetic strength or power, with both groups demonstrating small decrements after the training period (Table 2). This observation is inconsistent (and surprising) with previous results from our lab [23] given the significant improvement in leg press performance (Figure 2). All testing for each subject was performed in the same order during the pre- and post-testing sessions, yet the possibility exists that subjects may have been more fatigued from the 1RM testing during the post-training tests compared to the pre-testing sessions.

Limitations to the present study include: 1) diet, which can have a significant impact on the adaptations to resistance training [1,3,24], was not controlled, and 2) by design, the sample consisted of untrained subjects, and whether SS would be more effective at producing strength gains in more advanced athletes, where tissue repair may be more important compared to novice exercisers, cannot be addressed.

## Conclusions

Many supplements are commercially available; however, these supplements are often promoted without conclusive research demonstrating their efficacy. A recent review of 250 commercially advertised supplements found only 6 had been examined in randomized, placebo-controlled studies greater than 3 weeks in duration [5]. The present study demonstrates that twelve weeks of resistance training results in significant improvements in most measures of muscle strength and function, but the SS supplement did not lead to improvements above strength training alone.

## Competing interests

The authors declare that they have no competing interests. The study was funded in part by an urestricted gift to the Curry School of Education Exercise Physiology Fund from StemTech International, Inc. San Clemente, CA. FIK, JH, and AW served as scientific consultants for StemTech International.

## Author’s contributions

JF, CAR, JH, FIK, and AW contributed to the study conception and design, JF and MS acquired the data, JP performed the data analysis, JF, CAR, JH, FIK, and AW interpreted the data; All authors were involved in drafting the manuscript and have given final approval of the published version.

## References

[B1] Roy BD Tarnopolsky MA Influence of differing macronutrient intakes on muscle glycogen resynthesis after resistance exercise J Appl Physiol 1998 84 890 6 9480948 10.1152/jappl.1998.84.3.890

[B2] Conley MS Stone MH Carbohydrate ingestion/supplementation or resistance exercise and training Sports Med 1996 21 7 17 10.2165/00007256-199621010-00002 8771282

[B3] Biolo G Tipton KD Klein S Wolfe RR An abundant supply of amino acids enhances the metabolic effect of exercise on muscle protein Am J Physiol 1997 273 E122 9 9252488 10.1152/ajpendo.1997.273.1.E122

[B4] Tipton KD Ferrando AA Phillips SM Doyle D Jr Wolfe RR Postexercise net protein synthesis in human muscle from orally administered amino acids Am J Physiol 1999 276 E628 34 10198297 10.1152/ajpendo.1999.276.4.E628

[B5] Nissen SL Sharp RL Effect of dietary supplements on lean mass and strength gains with resistance exercise: a meta-analysis J Appl Physiol 2003 94 651 9 12433852 10.1152/japplphysiol.00755.2002

[B6] Baechle TRE Roger W Essentials of Strength Training and Conditioning 2001 Champaign, IL: Human Kinetics

[B7] Gribble PA Hertel J Plisky P Using the star excursion balance test to assess dynamic postural-control deficits and outcomes in lower extremity injury: a literature and systematic review J Athl Train 2012 47 339 57 22892416 10.4085/1062-6050-47.3.08 PMC3392165

[B8] StemSport® Advanced Formula http://www.stemtechbiz.com/StemSport.aspx

[B9] Jensen GS Hart AN Zaske LA Drapeau C Gupta N Schaeffer DJ Cruickshank JA Mobilization of human CD34+ CD133+ and CD34+ CD133(−) stem cells in vivo by consumption of an extract from Aphanizomenon flos-aquae–related to modulation of CXCR4 expression by an L-selectin ligand? Cardiovasc Revasc Med 2007 8 189 202 10.1016/j.carrev.2007.03.004 17765649

[B10] Shytle DR Tan J Ehrhart J Smith AJ Sanberg CD Sanberg PR Anderson J Bickford PC Effects of blue-green algae extracts on the proliferation of human adult stem cells in vitro: a preliminary study Med Sci Monit 2010 16 BR1 5 20037479

[B11] Weecharangsan W Opanasopit P Sukma M Ngawhirunpat T Sotanaphun U Siripong P Antioxidative and neuroprotective activities of extracts from the fruit hull of mangosteen (Garcinia mangostana Linn.) Med Princ Pract 2006 15 281 7 10.1159/000092991 16763395

[B12] Pilarski R Zielinski H Ciesiolka D Gulewicz K Antioxidant activity of ethanolic and aqueous extracts of Uncaria tomentosa (Willd.) DC J Ethnopharmacol 2006 104 18 23 10.1016/j.jep.2005.08.046 16202551

[B13] Purdy Lloyd KL Wasmund W Smith L Raven PB Clinical Effects of a Dietary Antioxidant Silicate Supplement, Microhydrin((R)), on Cardiovascular Responses to Exercise J Med Food 2001 4 151 9 10.1089/109662001753165738 12639409

[B14] Goud VK Polasa K Krishnaswamy K Effect of turmeric on xenobiotic metabolising enzymes Plant Foods Hum Nutr 1993 44 87 92 10.1007/BF01088486 8332589

[B15] Sumi H Hamada H Nakanishi K Hiratani H Enhancement of the fibrinolytic activity in plasma by oral administration of nattokinase Acta Haematol 1990 84 139 43 10.1159/000205051 2123064

[B16] Kubo M Asano T Shiomoto H Matsuda H Studies on rehmanniae radix. I. Effect of 50% ethanolic extract from steamed and dried rehmanniae radix on hemorheology in arthritic and thrombosic rats Biol Pharm Bull 1994 17 1282 6 10.1248/bpb.17.1282 7841954

[B17] Bloomer RJ Falvo MJ Schilling BK Smith WA Prior exercise and antioxidant supplementation: effect on oxidative stress and muscle injury J Int Soc Sports Nutr 2007 4 9 10.1186/1550-2783-4-9 17915021 PMC2131751

[B18] Bobeuf F Labonte M Dionne IJ Khalil A Combined effect of antioxidant supplementation and resistance training on oxidative stress markers, muscle and body composition in an elderly population J Nutr Health Aging 2011 15 883 9 10.1007/s12603-011-0097-2 22159777 PMC12878122

[B19] Ristow M Zarse K Oberbach A Kloting N Birringer M Kiehntopf M Stumvoll M Kahn CR Bluher M Antioxidants prevent health-promoting effects of physical exercise in humans Proc Natl Acad Sci U S A 2009 106 8665 70 10.1073/pnas.0903485106 19433800 PMC2680430

[B20] Krentz JR Quest B Farthing JP Quest DW Chilibeck PD The effects of ibuprofen on muscle hypertrophy, strength, and soreness during resistance training Appl Physiol Nutr Metab 2008 33 470 5 10.1139/H08-019 18461099

[B21] Novak ML Billich W Smith SM Sukhija KB McLoughlin TJ Hornberger TA Koh TJ COX-2 inhibitor reduces skeletal muscle hypertrophy in mice Am J Physiol Regul Integr Comp Physiol 2009 296 R1132 9 10.1152/ajpregu.90874.2008 19176887 PMC4043321

[B22] Flann KL LaStayo PC McClain DA Hazel M Lindstedt SL Muscle damage and muscle remodeling: no pain, no gain? J Exp Biol 2011 214 674 9 10.1242/jeb.050112 21270317

[B23] Dannelly BD Otey SC Croy T Harrison B Rynders CA Hertel JN Weltman A The effectiveness of traditional and sling exercise strength training in women J Strength Cond Res 2011 25 464 71 10.1519/JSC.0b013e318202e473 21217529

[B24] Willoughby DS Stout JR Wilborn CD Effects of resistance training and protein plus amino acid supplementation on muscle anabolism, mass, and strength Amino Acids 2007 32 467 77 10.1007/s00726-006-0398-7 16988909

